# Utility of contrast-enhanced computed tomography in the evaluation of canine insulinoma location

**DOI:** 10.1080/01652176.2018.1481545

**Published:** 2018-06-05

**Authors:** Floryne O. Buishand, Federico R. Vilaplana Grosso, Jolle Kirpensteijn, Sebastiaan A. van Nimwegen

**Affiliations:** a Department of Clinical Sciences of Companion Animals, Faculty of Veterinary Medicine, Utrecht University, Utrecht, The Netherlands; b Department of Diagnostic Imaging, College of Veterinary Medicine, University of Florida, Gainesville, FL, USA; c Global Veterinary and Professional Affairs, Hills Pet Nutrition, Topeka, KS, USA

**Keywords:** Canine, dog, insulinoma, CT, location sensitivity

## Abstract

**Objectives:** To determine 1) the sensitivity of contrast-enhanced CT (CECT) for detection of primary canine insulinomas and metastases 2) the sensitivity of CECT to locate canine insulinomas within the pancreas and 3) the CECT attenuation pattern of canine insulinomas and post-contrast phase in which insulinomas have the best visibility.

**Methods:** A retrospective review was performed of the medical records of 27 canine insulinoma patients. Simultaneous occurrence of blood glucose < 3.5 mmol/L (reference interval: 4.2–5.8 mmol/L) and plasma insulin > 10 mIU/L (reference interval: 1.4–24.5 mIU/L) were considered diagnostic for insulinoma. The dogs had a mean age of 9.0 ± 1.7 (SD) years and comprised 11 males and 17 females.

**Results:** Using CECT-scans, 26/27 insulinomas were successfully detected. However, CECT-scans predicted the correct location of insulinomas within the pancreas in only 14/27 dogs. In 9/13 inaccurately located insulinoma cases, the location error was major. There was no significant difference between triple, double and single-phase CECT-scans with location accuracies of 54%, 50% and 50%, respectively. Also, there was no specific post-contrast phase in which insulinomas could be visualised best. Detection of lymph node metastases with CECT-scans had a sensitivity of 67% (10/15 lymph node metastases). Detection of liver metastases had a sensitivity of 75% (6/8 liver metastases). This study highlights that major location errors mainly occurred if single- or double-phase CECT-scans were used (6/9 cases).

**Conclusion:** It is suggested that triple-phase CECT-scans have superior outcome over single- or double-phase CECT-scans in pre-operative imaging of canine insulinomas.

## 1. Introduction

Canine insulinomas are uncommon tumours of the pancreatic beta-cells that autonomously produce insulin, leading to hypoglycaemia (Madarame et al. 2009). Although canine insulinomas are rare, they are the most common malignant pancreatic endocrine tumours in the dog (Tobin et al. 1999). At time of diagnosis, 40%–50% of the insulinomas have already metastasised either to the regional lymph nodes or the liver (Leifer et al. 1986; Trifonidou et al. 1998). Based on survival analysis, surgical resection of the primary insulinoma and metastases, followed by medical therapy if required, is considered to be the better treatment option compared to medical therapy alone (Tobin et al. 1999; Polton et al. 2007).

Use of diagnostic imaging techniques, including transabdominal ultrasonography, contrast-enhanced ultrasonography, computed tomography (CT), single-photon emission computed tomography and somatostatin receptor scintigraphy, have been reported in the identification and pre-operative staging of canine insulinomas (Lamb et al. 1995; Garden et al. 2005; Robben et al. 2005; Nakamura et al. 2015). CT has proven to be the most sensitive method and has become the imaging modality-of-choice in pre-operative staging of canine insulinomas. Conventional CT has reported to have a sensitivity of 71% in detecting canine insulinomas. However, improved dual- or triple phase contrast-enhanced CT (CECT) correctly identified canine insulinomas in all 12 dogs from two different studies (Mai and Caceres 2008; Fukushima et al. 2016).

Pre-operative staging of insulinomas using diagnostic imaging techniques is of utmost importance. First of all, it provides valuable prognostic information as dogs with insulinomas with distant metastases have shorter survival times than dogs where the insulinoma is restricted to the pancreas and regional lymph nodes. Diagnostic imaging can also provide detailed information on the location of the insulinoma within the pancreas, which is important in surgical planning for insulinoma resection. Tumours located in the distal pancreatic lobes are often easily resected, while those located in the pancreatic body and closely associated proximal portions of the pancreatic lobes pose more of a surgical challenge to prevent damage to the pancreatic ducts and the pancreaticoduodenal arteries (Buishand and Kirpensteijn 2016). When considering laparoscopic excision of a canine insulinoma, it is even more important to determine the exact location of the insulinoma as it affects the decision to perform an open or laparoscopic surgical approach (Buishand et al. 2015). Sternal recumbency provides good laparoscopic access to the right lobe and the distal part of the left pancreatic lobe when a right or left-sided flank approach is used, but it is challenging or even impossible to reach the proximal left lobe or the corpus. In dorsal recumbency, the corpus and proximal part of the left lobe can be visualised more easily than the distal pancreatic lobe, because moving the omentum and stomach cranially becomes more difficult further to the left side. Therefore, the ideal diagnostic imaging technique for insulinomas should be able to distinguish the exact insulinoma location either in the distal, proximal lobes or pancreatic body, in order to allow optimal pre-operative planning.

Although CECT has proven to be very sensitive in diagnosing canine insulinomas (Mai and Caceres 2008; Fukushima et al. 2016), no data are available on the sensitivity of CECT for the location of canine insulinomas within the pancreas. Also, the sensitivity and specificity of diagnosing both primary insulinomas and metastases using CT-scans have only been analysed in a small number of dogs (Robben et al. 2005). Likewise, the largest case series describing the attenuation pattern of CECT only included nine dogs and demonstrated heterogenous enhancement patterns (Fukushima et al. 2016). Therefore, the goals of this study are to determine 1) the sensitivity of CECT for detection of primary canine insulinomas and metastases 2) the sensitivity of CECT for the exact location of canine insulinomas within the pancreas, and 3) the CECT attenuation pattern of canine insulinomas and post-contrast phase in which insulinomas have the best visibility.

## 2. Materials and methods

### 2.1. Cases

A retrospective review was performed of the medical records of canine insulinoma patients presented at the Department of Clinical Sciences of Companion Animals, Faculty of Veterinary Medicine, Utrecht University between 2005 and 2015. Dogs underwent single-, double-, or triple-phase CT, and data were available for evaluation. Insulinomas were surgically removed by an ECVS board certified surgeon (SvN or JK) using a LigaSure™ small jaw open instrument or LigaSure™ V, 5 mm shaft diameter (Covidien/Medtronic B.V., Heerlen, The Netherlands), as previously described (Wouters et al. 2011). Precise location of primary insulinoma (in the pancreatic body, proximal or distal half of the left or right lobe) was documented and the organ palpated for other masses. Furthermore, the abdominal lymph nodes and liver were also inspected and palpated for masses. Intra-operative glucose measurements were recorded demonstrating that normoglycaemia had returned after insulinoma and/or metastases resection, to assure that resection of macroscopic disease was complete. All insulinomas and metastases were confirmed by histological examination.

Intra-operative locations were based on standard anatomical landmarks. The pancreatic body was defined as the area where the left and right pancreatic lobes unite and where the cranial pancreaticoduodenal artery and gastroduodenal vein enter the pancreas. The descending duodenum was used as an anatomical landmark for the right pancreatic lobe and the dorsal extremity of the spleen and the gastric fundus were used as landmarks for the left lobe. The proximal half of a pancreatic lobe was identified as the half of the lobe closest to the pancreatic body. Similarly, the distal half was the part of the lobe located most distal to the pancreatic body.

Clinico-pathological characteristics of insulinomas included in this study are summarised in Table 1. The simultaneous occurrence of blood glucose < 3.5 mmol/L (reference interval (RI): 4.2–5.8 mmol/L) and plasma insulin > 10 mIU/L (RI: 1.4–24.5 mIU/L) were considered diagnostic for insulinoma (Fernandez et al. 2009). Twenty-seven dogs could be included in the study. Five dogs underwent a second CECT-scan because of reoccurence of hypoglycaemia after partial pancreatectomy and one dog underwent a third CECT-scan because of relapse of clinical signs after a second treatment, rendering a total of 33 CECT-scans (Table 2).

**Table 1.** General characteristics of the 27 dogs and the corresponding insulinoma.

Dog	Breed	Sex	Body weight (kg)	Body surface area (m^2^)	Age (years)	Pre-operative glucose (mmol/L)	Pre-operative insulin (mIU/L)	INS diameter (cm)	TNM stage^a^
1	Maltese	FC	4.8	0.29	11	3.2	38	1.0	III
2	Jack Russell terrier	FC	6.5	0.35	12	2.4	11.2	1.0	I
3	Boxer	M	44.8	1.27	8	3.0	46	1.0	IV
4	Crossbred	MC	23.2	0.82	10	3.0	39	1.0	I
5	Irish Softcoated Wheaten terrier	MC	20.8	0.76	12	3.4	31	1.2	I
6	Crossbred	MC	33.1	1.04	8	3.3	–	1.5	IV
7	West Highland white terrier	FC	6.7	0.36	10	3.6	37.2	1.3	I
8	Labrador Retriever	M	30.7	0.99	8	2.8	15	1.0	I
9	Braque Francais	FC	21.2	0.77	8	2.7	13	2.0	II
10	Border Collie	F	20.9	0.77	7	3.0	18	0.5	I
11	Field Spaniel	M	26.1	0.89	9	1.8	107	1.5	IV
12	Flatcoated Retriever	M	37.8	1.14	7	2.9	25	2.5	II
13	West Highland white terrier	FC	7.6	0.39	11	1.6	64	1.5	IV
14	Boxer	MC	34.5	1.07	8	2.8	16	2.0	II
15	Jack Russell terrier	FC	3.2	0.22	9	2.4	111	2.0	III
16	Jack Russell terrier	FC	9.2	0.44	11	2.4	29	1.5	I
17	Basset Artesien Normand	FC	19.6	0.73	9	3.0	18	3.0	III
18	Yorkshire terrier	MC	4	0.26	8	2.7	17	1.0	I
19	Kooiker dog	F	10.7	0.49	8	3.1	96	1.5	III
20	Boxer	FC	36.9	1.12	11	3.1	31	3.0	III
21	Jack Russell terrier	M	12	0.53	8	2.3	–	1.0	IV
22	Crossbred	FC	31.1	1.00	6	2.4	–	0.5	I
23	Bearded Collie	F	20.5	0.76	9	3.3	–	0.5	IV
24	Dachshund	FC	9.9	0.47	8	2.8	14	1.3	III
25	Crossbred	MC	41.8	1.22	7	2.8	16	1.0	I
26	Scottish Shepherd dog	F	29.9	0.97	8	1.9	34	4.0	IV
27	German Pointer	FC	22.5	0.81	11	2.5	29.7	3.0	II

INS, insulinoma; F, female; M, male; FC, neutered female; MC, neutered male; ND, not determined.

a Staging was performed according to Buishand et al. (2010).

**Table 2.** CT findings in dogs with insulinoma.

Dog	Type CT-scan	Best visibility (phase)	CT INS location	Surgery INS location	CT lymph node status	Surgery lymph node status	CT liver status	Surgery liver status
1	Triple-phase	Same all phases	Body	Body	N1	N1	M0	M0
2	Triple-phase	Portal	L2	L1	N0	N0	M0	M0
3a	Triple-phase	Arterial	R1	R1	N1	N1	M0	M0
3b	Double-phase	–	–	–	N0	N1	M0	M0
3c	Double-phase	–	–	–	N1	N1	M0	M1
4	Triple-phase	Portal	L2	L2	N0	N0	M0	M0
5	Triple-phase	Arterial	L2	L2	N1	N0	M1	M0
6	Triple-phase	Arterial	Body	L1	N1	N1	M1	M1
7	Triple-phase	Arterial	L2	L1	N0	N0	M0	M0
8	Triple-phase	Portal	L2	L2	N0	N0	M0	M0
9	Triple-phase	Portal	R1	R1	N0	N0	M0	M0
10	Triple-phase	Same all phases	R2	R2	N0	N0	M0	M0
11	Triple-phase	Same all phases	L2	L1	N1	N1	M1	M1
12	Triple-phase	Arterial	L1	Body	N0	N0	M0	M0
13	Triple-phase	–	No lesion	L2	N0	N0	M1	M1
14	Double-phase	Arterial	L1	L1	N0	N0	M1	M0
15a	Double-phase	Arterial	L1	L1	N0	N0	M0	M0
15b	Double-phase	–	–	–	N0	N1	M1	M1
16	Double-phase	Arterial	L2	L2	N0	N0	M1	M0
17	Double-phase	Arterial	R1 + L1	Body	N1	N1	M0	M0
18	Double-phase	Portal	L2	L2	N0	N0	M0	M0
19a	Double-phase	Same all phases	Body	R2	N0	N1	M0	M0
19b	Single-phase	–	–	–	N0	N1	M0	M0
20	Double-phase	Portal	Body	L2	N0	N1	M1	M0
21	Double-phase	Delayed	L2	L1	N1	N0	M0	M1
22	Double-phase	Portal	R1	L2	N0	N0	M0	M0
23a	Double-phase	Portal	R2	R2	N1	N1	M0	M0
23b	Double-phase	–	–	–	N1	N1	M1	M1
24a	Single-phase	Delayed	Body	R1	N0	N0	M0	M0
24b	Double-phase	–	–	–	N1	N1	M0	M0
25	Single-phase	Delayed	R2	L2	N0	N0	M0	M0
26	Single-phase	Portal	R2	R2	N1	N1	M1	M1
27	Single-phase	Arterial	Body	Body	N0	N0	M0	M0

L1, proximal left pancreatic lobe; L2, distal left pancreatic lobe; R1, proximal right pancreatic lobe; R2, distal right pancreatic lobe; N0, no evidence of lymph node metastasis; N1, lymph nodes involved; M0, no evidence of liver metastasis; M1, liver metastasis present.

### 2.2. Computed tomography

All dogs were classified as ASA 3 based on the ASA physical status classification system of the American Society of Anesthesiologists (Pierce 1990). Dogs were premedicated with 0.5 mg/kg BW IM methadone; anaesthesia was induced with 4–6 mg/kg BW IV propofol and maintained with 5–20 μg/kg BW/hour IV fentanyl and isoflurane in 100% oxygen delivered through a cuffed endotracheal tube. After induction of general anaesthesia, dogs were positioned in dorsal (*n* = 31 studies) or ventral recumbency (*n* = 2 studies) on the CT table using a single-slice CT scanner (Philips Secura, Philips NV, Eindhoven, The Netherlands) (*n =* 26 dogs / 32 studies) or a 64-slice CT scanner (Siemens SOMATOM Definition AS, Siemens Nederland, Den Haag, The Netherlands) (*n* = 1 dog / 1 study). Short-term apnea was induced with manual hyperventilation for all dogs. Scans were made in helical acquisition mode with a slice thickness of 2–7 mm and a pitch of 1–1.5, depending on patient size. The kVp and mA were not standardised. The field-of-view was selected to include the entire abdomen. Other technical settings were 0.7–1 sec tube rotation time, a reconstruction index of 0.5–1, 512 × 512 matrix, and a medium frequency reconstruction algorithm.

After evaluation of the pre-contrast series, Iobitridol (Xenetix® 350, Guerbet Nederland BV, Gorinchem, The Netherlands) was administered at a dose of 700 mg IV/kg BW delivered via the cephalic vein using an automatic angiographic injection system at a fixed injection rate of 5 mL/s (Medrad® Mark V plus, Medrad Europe B.V., Beek, The Netherlands). Dogs underwent either a single-phase CT (*n* = 5), a double-phase CT (*n* = 15), or a triple-phase CT (*n =* 14), resulting in a total of 22 arterial phases, 27 portal phases and 25 delayed phases. These phases were acquired approximately 15 sec, 30 sec, and 90 sec after injection of contrast media, respectively, as described previously (Iseri et al. 2007).

### 2.3. Computed tomography image analysis

The CT images were retrospectively reviewed by one board-certified radiologist (FVG) blinded to intra-operative findings. Images were reviewed in a random order using image analysis freeware (OsiriX v.5.8.2 32-bit, Pixmeo, Geneva, Switzerland). Display settings were adjusted as needed for optimal evaluation of the images. Primary insulinomas were identified as mass lesions in the pancreas and location and size were evaluated. In analogy to the classification that was used during surgery, insulinoma location was classified in either one of the following locations, using the anatomical landmarks that were used during surgery: pancreatic body, left or right proximal lobe, or left or right distal lobe. The CT phase during which the insulinoma subjectively displayed the best visibility was recorded. Furthermore, presence or absence of lymphadenomegaly and hepatic nodular changes were recorded.

Attenuation of masses was measured using the maximum circular region of interest (ROI) that could be fitted to each mass. Identical ROIs were placed in the same location in pre-contrast and all post-contrast images. In the post-contrast images, hyper and hypo-attenuation were defined respectively as a positive or negative difference of at least 20 Hounsfield units (HU) between the mass and the surrounding normal pancreatic parenchyma. Iso-attenuation was defined as attenuation within 20 HU of the normal pancreatic parenchyma.

## 3. Statistical analysis

The student's *t*-test was used to compare the average age, body weight, body surface area and tumour size of dogs with inaccurately located insulinomas versus accurately located insulinomas. A chi-squared (χ^2^) test was used to assess the difference in insulinoma location and CECT-scan type between the inaccurately versus accurately located insulinomas. A *P*-value of 0.05 was considered to indicate a significant difference. Statistical analyses were performed using Prism 7 (GraphPad Software, La Jolla, USA).

## 4. Results

The included dogs had a mean age of 9.0 ± 1.7 (SD) years and comprised 11 males and 16 females. The predominant breeds were Jack Russell terrier (*n* = 4) and crossbred (*n* = 4).

**Figure 1.**
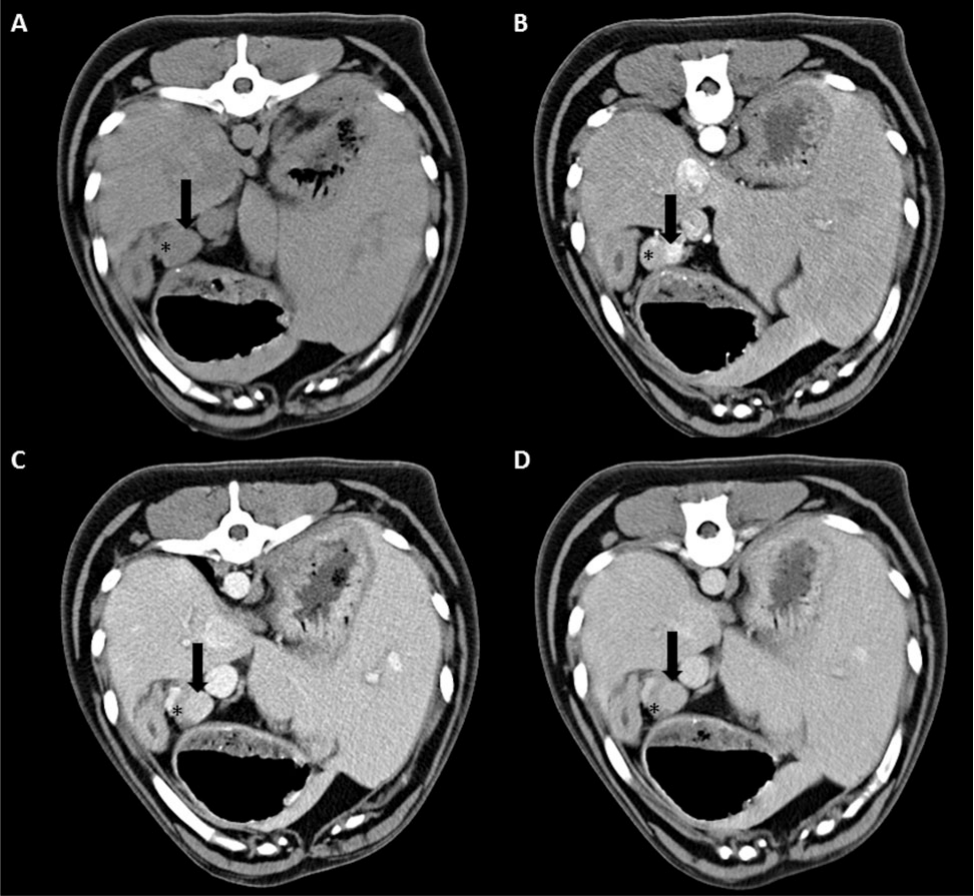
Transverse triple-phase contrast-enhanced computed tomography (CECT) of a 7-year-old male Flatcoated Retriever (case 12) with insulinoma in the pancreatic body (black arrow). A nodule is noted deforming the contours of the pancreas. This nodule is very mildly hypo-attenuating compared to the adjacent pancreatic parenchyma (asterisk) on pre-contrast images (A). The pancreatic nodule is strongly hyper-attenuating on the arterial phase (B) and remains hyper-attenuating on the portal phase (C). The delayed phase demonstrates iso-attenuation of the pancreatic nodule (D).

Detection of primary insulinomas with CECT-scans had a sensitivity of 96% (26/27 insulinomas). The location sensitivity was 52%, because CECT-scans predicted the correct location of insulinoma within the pancreas in 14/27 dogs (Table 2). Triple-phase, double-phase and single-phase CECT-scans had location sensitivities of 54% (7/13 insulinomas), 50% (5/10 insulinomas) and 50% (2/4 insulinomas), respectively. Ten insulinomas had the best visibility on CECT-scans during the arterial phase, nine insulinomas had best visibility during the portal phase, three insulinomas during the delayed phase and in four cases insulinomas had the same visibility in all phases (Table 2). Student's *t*-tests and χ^2^ tests demonstrated that age, body weight, body surface area, tumour size, insulinoma location and CECT type were not significantly different between dogs with inaccurate versus accurately located insulinomas (Table 3). The overall attenuation of insulinomas on CECT-scans is summarised in Table 4. Lesions were either iso- or hypo-attenuating before contrast administration, with iso-attenuation being the most common pattern (20/26 insulinomas). After contrast administration, iso-, hypo-, and hyper-attenuation were seen in all phases of the CECT-scans. An example of an insulinoma that demonstrated iso-, hypo-, and hyper-attenuation in the different phases is presented in Figure 1. Iso- and hyper-attenuation were most common in the arterial phase (both 8/17 insulinomas). Iso-attenuation was the most common pattern in both the portal phase (16/23 insulinomas) and the delayed phase (11/20 insulinomas). Insulinomas that were not correctly located on CECT-scans did not have a distinctive attenuation pattern in comparison to insulinomas that were correctly located.

**Table 3.** Contrast-enhanced CT outcomes according to variables.

Variable	Inaccurately located cases (*n* = 13)	Accurately located cases (*n* = 14)	*P*-value
	Mean±SD	Mean±SD	
**Age**	8.7 ± 1.8	9.1 ± 1.6	0.55^a^
**Weight**	21.5 ± 13.4	20.7 ± 12.2	0.87^a^
**Body surface area**	0.75 ± 0.33	0.73 ± 0.32	0.88^a^
**Tumour size**	1.6 ± 0.8	1.6 ± 1.0	1.00^a^
	Number of cases	Number of cases	
**Tumour location**			0.61^b^
Body	2	2	
R1	1	2	
R2	1	3	
L1	5	2	
L2	4	5	
**CT-scan type**			0.98^b^
Single-phase	2	2	
Double-phase	5	5	
Triple-phase	6	7	

L1, proximal left pancreatic lobe; L2, distal left pancreatic lobe; R1, proximal right pancreatic lobe; R2, distal right pancreatic lobe.

a Student's *t-*test

b χ^2^*-*test

**Table 4.** Canine insulinoma attenuation patterns.

Dog	Accurate CT insulinoma location	Non-contrast	Arterial	Portal	Delayed
1	Yes	Iso	Iso	Iso	Hyper
2	No	Iso	Hyper	Iso	Hyper
3	Yes	Iso	Iso	Hyper	Iso
4	Yes	Iso	Iso	Hyper	Hyper
5	Yes	Hypo	Hyper	Hyper	Hypo
6	No	Iso	Iso	Iso	Iso
7	No	Hypo	Hyper	Iso	Iso
8	Yes	Iso	Iso	Hyper	Hyper
9	Yes	Hypo	Iso	Iso	Iso
10	Yes	Iso	Hypo	Iso	Iso
11	No	Iso	Iso	Iso	Iso
12	No	Hypo	Hyper	Iso	Hypo
13	No	–	–	–	–
14	Yes	Iso	Hyper	Iso	–
15	Yes	Iso	Hyper	Iso	–
16	Yes	Hypo	Hyper	Hyper	–
17	No	Iso	Iso	Hypo	–
18	Yes	Iso	–	Iso	Iso
19	No	Iso	–	Iso	Iso
20	No	Iso	–	Iso	Iso
21	No	Iso	–	Iso	Hypo
22	No	Iso	–	Iso	Hyper
23	Yes	Iso	–	Iso	Iso
24	No	Iso	–	–	Hyper
25	No	Iso	–	–	Iso
26	Yes	Hypo	–	Hyper	–
27	Yes	Iso	Hyper	–	–

In 4/13 inaccurately located insulinomas the location error was minor, all insulinomas predicted in the distal left lobe based on CECT-scans were located in the proximal left lobe. However, the predicted location of the remaining tumours was highly inaccurate. Two of these tumours were predicted to be in a lobe and found in the body, while another four tumours, predicted in the body were found in either right or left lobe. Another two tumours predicted in the right lobe were found in the left lobe and a CECT undetected insulinoma was only located during surgery in the distal left pancreatic lobe.

**Figure 2.**
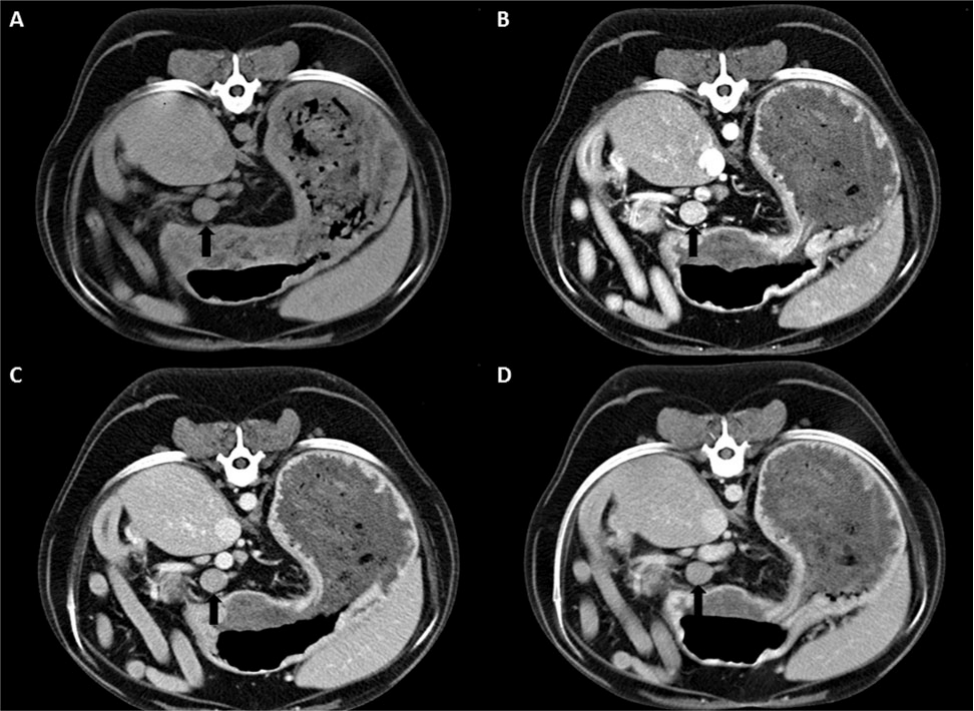
Transverse triple-phase contrast-enhanced computed tomography (CECT) in an 8-year-old neutered male crossbred (case 6) with metastatic insulinoma lymphadenopathy. The pancreaticoduodenal lymph node (black arrow) is enlarged and round. The lymph node is homogenously iso-attenuating on pre-contrast images (A) and all post-contrast phases (arterial (B), portal (C) and delayed venous (D)), demonstrate hyper-attenuation.

**Figure 3.**
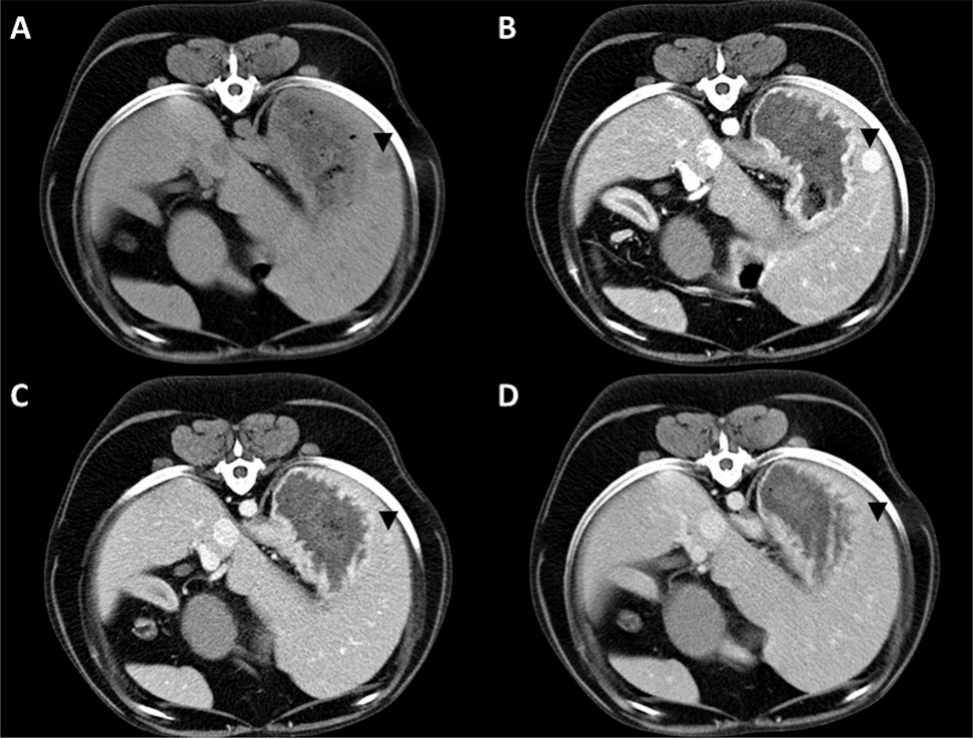
Transverse triple-phase contrast-enhanced computed tomography (CECT) in an 8-year-old neutered male crossbred (case 6) with a hepatic insulinoma metastasis. A well-defined nodule is noted in the periphery of the left lateral hepatic lobe (black arrow head). The nodule is hypo-attenuating on pre-contrast images (A). During the arterial phase the nodule shows homogeneous strong enhancement (B). The center of the nodule remains hyper-attenuating during the portal and delayed venous phases with a less attenuating periphery (C and D).

Detection of lymph node metastases (Figure 2) with CECT-scans had a sensitivity of 67%, since 10 out of 15 metastatic lymph nodes were identified on CECT. Of the CECT-scans that predicted an inaccurate lymph node status, two were triple-phase CECTs, four were double-phase CECTs and one CECT-scan only included the delayed phase. Detection of liver metastases (Figure 3) with CECT-scans had a sensitivity of 75% (6/8 liver metastases). Of the CECT-scans that predicted an inaccurate liver status, one was a triple-phase CECT and five were double-phase CECTs.

## 5. Discussion

This study is the first report on detection and location sensitivity of a case series of CECT-scans of canine insulinomas. Despite the high sensitivity (96%) of CECT in detecting canine insulinoma, the location sensitivity was only 52%. A similar location sensitivity of 55% of CECT-scans was recently reported in a study including 31 human insulinomas (Nockel et al. 2017). Another study, including 47 human patients with insulinomas, divided over three groups, also reported an overall location sensitivity of CECT of 57.4%, with a range of 35.3–81.3% depending on the scanning protocol that was used (Long et al. 2009). One of the main reasons for the low location sensitivity is that both canine and human insulinomas demonstrate very heterogeneous enhancement patterns. These patterns make the interpretation of the diagnostic images hard because a well-defined specific scoring pattern is lacking. Additionally, this study pointed out that there is no specific post-contrast phase in which insulinomas could be visualised best. It seemed that insulinomas have the best visibility in the arterial phase (10/26 insulinomas; 38%) demonstrating either iso-, or hyper-attenuation. This is in contrast to an earlier study that reported hypo-attenuation to be the most common pattern in the arterial phase of CECT-scans of canine insulinomas (Fukushima et al. 2016). That study only included nine canine insulinoma cases, of which four demonstrated hypoattenuation, three iso-attenuation, and two hyper-attenuation. Our results are more in line with the fact that hyper-attenuation has been reported as the most common enhancement pattern in the arterial phase of CECT-scans of human insulinomas (Fidler et al. 2003). Our 6% hypo-attenuation data is also in line with the percentage of 13% arterial phase hypo-attenuation in human insulinomas (Fidler et al. 2003). It remains unclear why insulinomas show such great variability in appearance on CECT-scans and more research focused on histological features of insulinomas, like vascularity and dispersion of connective tissue, is required to clarify the different CECT findings.

There was no overall difference between triple-phase, double-phase, and single-phase CECT-scans with location sensitivities of 54% 50%, and 50%, respectively. However, when we focused on only major location errors, triple-phase CECT performed better compared to double or single-phase CECTs, with major location error rates of 17% (2/12 triple-phase scans) versus 50% (5/10 double-phase scans) and 50% (2/4 single-phase scans). These major location errors have to be prevented during pre-operative planning, since these errors have the highest impact on the procedure of surgical insulinoma resection. For instance in cases 12 and 17, surgery was complicated because it was only noted intra-operatively that the insulinomas were located in the pancreatic body, while they were predicted to reside in the pancreatic lobes. On the other hand, it is interesting to note that out of 6 insulinomas that were predicted to be located in the pancreatic body, only 2 insulinomas were actually located in the pancreatic body. Pancreatic body location of an insulinoma may be a contra-indication for surgery, as depending on the exact location, there is a high risk of damage to the ductal system and the pancreaticoduodenal arteries during surgery (Buishand and Kirpensteijn  2016). CT may not be the ideal way of determining pancreatic body insulinoma because a CT-based prediction of a pancreatic body location is wrong in 67% of the cases reported here. The fact that insulinomas were predicted to be present in the right pancreatic lobe, but were intra-operatively detected in the left pancreatic lobe in cases 22 and 25 could have been due to the fact that the descending duodenum is not always an accurate anatomical landmark for the right pancreatic lobe. The position of the duodenum is strongly depending on its filling (Probst and Kneissl 2001), and in these cases the duodenum could have been located somewhat more to the left on CT, resulting in an aberrant prediction of the insulinomas in the right lobe, while they were actually located in the left lobe.

Magnetic Resonance Imaging (MRI) has been reported to correctly identify 29/31 (94%) pancreatic lesions in human patients with pancreatic neuroendocrine tumours, and has been suggested as the preferred imaging modality for human insulinomas (Owen et al. 2001). In veterinary medicine, MRI studies of the canine abdomen are inconsistent. However, this imaging technique is gaining popularity. Manley et al. (2013) evaluated different MRI protocols for their diagnostic quality regarding the canine abdomen using a 1.0 Tesla magnet. More recently the perfusion and diffusion characteristics of the liver in healthy dogs were evaluated using a 3.0 Tesla magnet (Del Chicca et al. 2016). On top of that, MRI has already proven to be superior to CT in diagnosing a canine mesenteric lymphoma and a large splenic tumour in a dog (Yasuda et al. 2004; Kim et al. 2016). Further research into the use of MRI for canine abdominal imaging is warranted. It is expected that MRI may prove to be better in differentiating between insulinoma locations (distal, mid, proximal lobes, and pancreatic body) compared to CECT-scans, which would facilitate pre-operative planning. There is a clear need for better pre-operative imaging modalities, since this would facilitate the use of laparoscopic pancreatectomy instead of traditional open surgery. In human medicine, the minimal invasive approach has been associated with similar or shortened surgery times, less blood loss, fewer complications, and decreased hospitalisation time compared to the open approach (Tang et al. 2007; Merchant et al. 2009). Currently, except for laparoscopic or laparoscopic-assisted pancreatic biopsies, the use of laparoscopic surgery of the pancreas in companion animal patients is not a standard procedure, and has only been reported in one dog with an insulinoma in the mid-left lobe of the pancreas (McClaran et al. 2017). Although MRI seems promising, drawbacks for the use of MRI for canine abdominal imaging could be the limited availability and prolonged image acquisition time compared to CT.

CECT-scans demonstrated sensitivities of 67% and 75% regarding detection of lymph node and liver metastases, respectively. There are no other case series including at least 27 patients, comparing the sensitivity of CECT-scans for the detection of either canine or human insulinoma metastases. However, these sensitivities are in line with those reported for other human abdominal epithelial tumours (Lee et al. 2009; Wang et al. 2013; Servaes et al. 2015; Schulz et al. 2016). Our case series included two cases that demonstrated abnormal lymph nodes on CECT-scans, but no lymphadenomegaly was found during surgery. Although it is unlikely that these were metastatic lymph nodes, we cannot exclude that these lymph nodes contained micrometastases, because only lymph nodes that were abnormal on palpation were sampled.

The present study has several limitations. First of all, this study was retrospective in nature and was conducted at an academic referral clinic, which might have resulted in a biased patient population, since the referring veterinarian may have treated the insulinomas that were easy to localise. Furthermore, although body weight and body surface area were not significantly associated with CECT location sensitivity, using the same contrast medium injection protocol for all dogs may have affected the results of the enhancement patterns. It takes more time for injection of the contrast medium in large breed versus small breed dogs. Therefore, to reduce the heterogeneity in enhancement patterns it might be advisable to use more precise methods, like a test injection or bolus-tracking method for canine pancreatic CECT imaging. Only one board certified veterinary radiologist scored the CECT images. The sensitivity, specificity and location sensitivity of the CECT-scans may be improved if multiple radiologists would score the CECT images. Although the radiologist was blinded to the intra-operative location of the insulinomas, he did know that all cases had insulinoma. This bias could explain why there were major location errors in some cases. In all cases a potential pancreatic insulinoma location was identified, even when the mass was not clearly visible in any of the contrast series. Other factors that could have contributed to a low overall location sensitivity is the use of a single-slice CT scanner for the majority of the patients and the use of varying slice thickness. The overall sensitivity, specificity and location sensitivity might improve if a 64-slice CT scanner with a standardised triple-phase CT protocol is used. Also, the use of poor anatomical landmarks could have played a role in the inaccurate location prediction of insulinomas with CT imaging, because of differences between patients in terms of location, size and position of the pancreas and the organs that were used as anatomical landmarks.

In conclusion, there was no difference in location sensitivities between triple-phase, double-phase and single-phase CECT-scans. However, major location errors mainly occurred if single- or double-phase contrast-enhanced CECT-scans were used (6/9 cases). Therefore, we suggest that triple-phase CECT-scans have superior outcome over single- or double-phase CECT-scans and future prospective studies in a mixed population of dogs with and without insulinomas are required to support this theory.
